# LINC00094/miR-19a-3p/CYP19A1 axis affects the sensitivity of ER positive breast cancer cells to Letrozole through EMT pathway

**DOI:** 10.18632/aging.204110

**Published:** 2022-06-02

**Authors:** Yuan Xiang, Hui Liu, Hao Hu, Le-Wei Li, Qi-Bei Zong, Tang-Wei Wu, Xiao-Yi Li, Shi-Qiang Fang, Yi-Wen Liu, Yu Zhan, Hui Wang, Zhong-Xin Lu

**Affiliations:** 1 Department of Medical Laboratory, Central Hospital of Wuhan, Tongji Medical College, Huazhong University of Science and Technology, 430014, Hubei, P.R. China; 2 Institute of Biology and Medicine, College of Life and Health Sciences, Wuhan University of Science and Technology, 430081, Hubei, P.R. China

**Keywords:** LINC00094, miR-19a-3p, endocrine therapy of breast cancer

## Abstract

The endocrine therapy resistance of breast cancer is the difficulty and challenge to be urgently solved in the current treatment. In this study, we examined the effects of noncoding RNA LINC00094 and miR-19a-3p on breast cancer *in vivo* and *in vitro* by RT-QPCR, Western Blot, luciferase assay, immunofluorescence and drug sensitivity tests. The plasma level of CYP19A1 in patients with breast cancer resistance was lower than that in drug sensitive patients. Compared with normal subjects, miR-19a-3p was highly expressed in plasma of patients with breast cancer. miR-19a-3p is highly expressed in estrogen receptor positive breast cancer cells. The expression of miR-19a-3p promoted the migration and EMT of breast cancer cells and reduced the sensitivity of breast cancer to Letrozole. LINC00094 sponge adsorbed miR-19a-3p. LINC00094 promotes the expression of CYP19A1, the target gene of miR-19a-3p, and inhibits the EMT process of breast cancer, ultimately promoting the sensitivity of ER-positive breast cancer cells to Letrozole. This study found a new mechanism of Letrozole sensitivity in ER positive breast cancer.

## INTRODUCTION

The most obvious change in the world’s latest cancer data in 2020 is the rapid growth of new cases of breast cancer, which has become the most common cancer in the world [1]. Among them, estrogen receptor (ER)-positive patients account for about 70% of all breast cancers, and endocrine therapy is usually the option for such patients. Although endocrine therapy and targeted therapy have good results in ER-positive patients, 20% of women still develop cancer recurrence or metastasis [2, 3]. Drug resistance is an important cause of the failure of endocrine therapy for breast cancer [4]. At present, the causes of endocrine therapy resistance include estrogen receptor α (ESR1) gene mutation, gene polymorphism of aromatase (CYP19A1), receptor tyrosine kinase (RTKs) and activation of downstream pathways [5]. The high incidence rate of breast cancer, the poor prognosis and resistance mechanism of endocrine therapy are not fully clear, which prompted us to further study and understand the mechanism of breast cancer endocrine therapy, and more importantly, to establish effective therapeutic targets for endocrine therapy.

For postmenopausal patients, aromatase inhibitors (AIs) are preferred for first-line endocrine therapy. It mainly works by inhibiting the activity of aromatase (CYP19A1), reducing the production of estrogen in the body. Despite the significant effect of AIs treatment, more than 20% newly diagnosed breast cancer patients will develop tumor progression or relapse within ten years of using the drug [6]. Letrozole belongs to non-steroidal AIs and has no potential toxicity to all systems and target organs of the whole body. Compared with other AIs and SERMs, Letrozole has stronger antitumor effect [7]. The mechanism of AIs resistance involves epithelial mesenchymal transition (EMT) [8], but the relationship between EMT and the sensitivity of breast cancer to Letrozole is still unclear.

Noncoding RNA, especially miRNAs and LncRNAs, are involved in regulating the therapeutic sensitivity of almost all treatments for breast cancer [9]. MiRNA is a class of noncoding RNA encoding 22 nucleotides, encoding endogenous genes. Our previous study reported that miR-206 is involved in breast cancer migration, and miR-93-5p regulates breast cancer mesenchymal transition (EMT). Let-7a-5p affects the glycometabolism of breast cancer and miR-17-5p can accelerate the apoptosis of breast cancer cells induced by paclitaxel [10–14]. Studies have shown that abnormal expression of miRNAs can lead to drug resistance in breast cancer. MiR-27a, miR-34a, miR-146a, miR-21 and miR-148a may be potential targets for tamoxifen resistance in breast cancer. Meanwhile, Zhou et al. [15] found that miR-3178, miR-1228, miR-138, miR-486-5p and miR-4532 are expected to be new targets for improving resistance to endocrine therapy. MiR-575, miR-182-5p, miR-18a and miR-449a promote the drug resistance of endocrine therapy for breast cancer [16–19]. What is interesting is that miR-19 not only affects the proliferation and metastasis of breast cancer [20–24], but also regulates the multidrug resistance of breast cancer [25]. However, the role of miR-19 in endocrine therapy resistance, especially in AIs treatment resistance, has not been reported.

LncRNAs are noncoding RNAs composed of transcripts of more than 200 nucleotides, which participate in the regulation of various physiological processes of breast cancer. We reported that LncRNA H19 can regulate the EMT of breast cancer [12]. LncRNA regulates breast cancer endocrine therapy resistance: LncRNA-UCA1 mediates Wnt / β-Catenin and AKT / mTOR signaling pathway promotes breast cancer endocrine therapy resistant [25]. LncRNA HOTAIR increased resistance to tamoxifen by promoting ER signal transduction [12]; LncRNA HOTAIRM1 promotes breast cancer drug resistance by promoting HOXA1 expression in breast cancer cells [26]. LncRNA Gas5 functions in the endogenous competitive RNA (competing endogenous RNAs, ceRNA) [27]. The expression of LINC00094 is associated with poor prognosis of lung squamous cell carcinoma [28]. Expression of LINC00094 has also been associated with esophageal squamous cell carcinoma and Alzheimer’s disease [29, 30]. However, the role of LINC00094 in endocrine resistance of breast cancer is currently unknown.

In our study, we found that LINC00094 sponge adsorbed miR-19a-3p, while miR-19a-3p was highly expressed in plasma and breast cancer cells of patients with breast cancer, and high expression of hsa-miR-19a-3p in breast cancer patients in LINC00094 group. The overall survival rate of these patients is very low. These findings provide new ideas for the clinical treatment of breast cancer.

## RESULTS

### CYP19A1 promotes ER positive breast cancer sensitivity to letrozole

**Figure 1:**
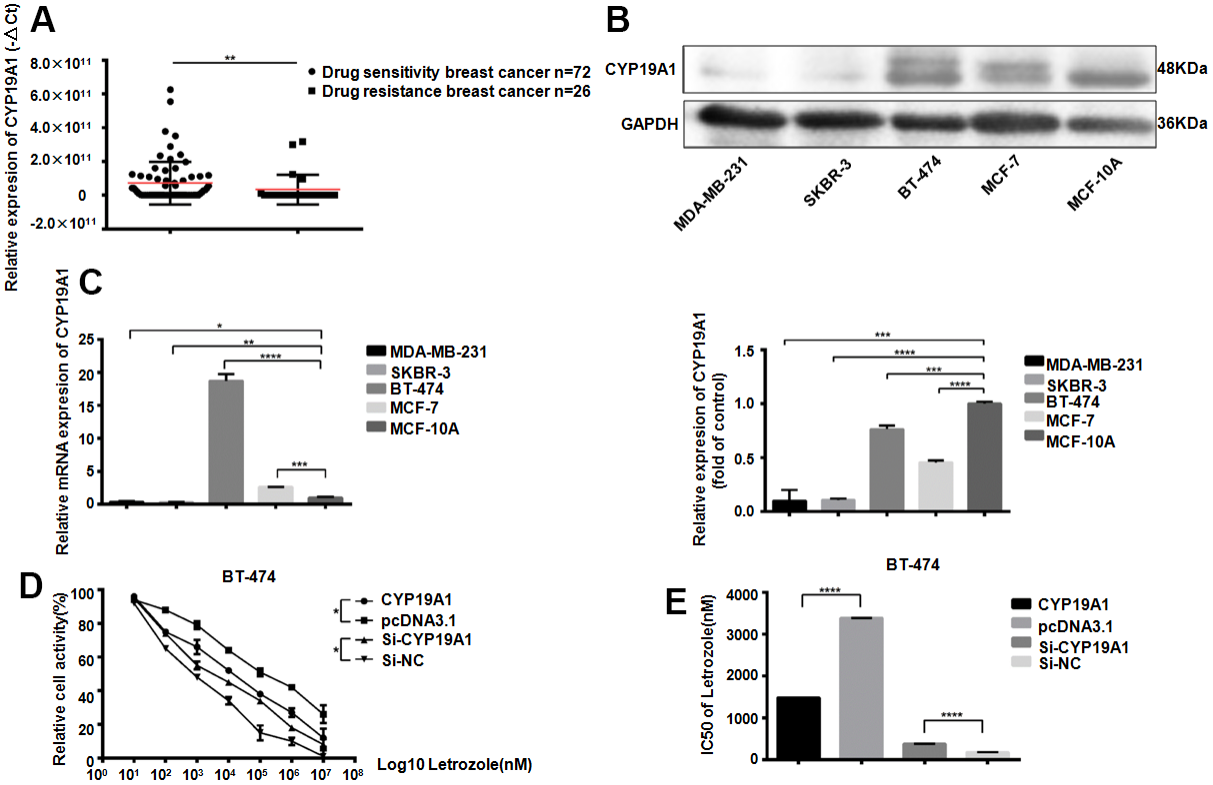
**CYP19A1 expression promotes the sensitivity of ER positive breast cancer cells to Letrozole.** (**A**) mRNA expression level of CYP19A1 in plasma of breast cancer patients was detected by RT-QPCR and analyzed by Mann Whitney test.**P<0.01. (**B**) Western Blot Analysis of endogenous protein expression levels of CYP19A1 in 5 breast cancer cell lines, using GAPDH as an internal reference, and 3 experiments were repeated. ***P<0.001, ****P<0.0001. (**C**) RT-QPCR was used to detect the endogenous mRNA expression level of CYP19A1 in 5 breast cancer cell lines. β-actin was used as an internal reference, and 3 replicates were carried out each time. *P<0.05, **P<0.01, ***P<0.001, ****P<0.0001. (**D**) BT-474 cells in 4 groups were treated with 8 concentrations of Letrozole, and CCK-8 was added 48 hours later to detect cell activity. Cells in each group had 3 repeated Wells. **P<0.01. (**E**) Graphpad Prism calculated the IC50 of four groups of BT-474 cells against Letrozole. ****P<0.0001.

The CYP19A1 gene encodes aromatase, which converts androstenedione and testosterone into estrogen. We first detected the CYP19A1 expression from 26 patients plasma with drug-resistant breast cancer, comparing with 72 patients with non-drug-resistant patients. The drug-resistant breast cancer patients CYP19A1 mRNA expression level was significantly downregulated (**P<0.01) (Figure 1A). Meanwhile, CYP19A1 is upregulated in the ER positive cell lines (MCF-7, BT-474) of all the 5 breast cancer cell lines, with the highest expression in BT-474, while almost no expression in ER negative breast cancer cell lines (Figure 1B, 1C). CCK-8 cell activity test showed that different level of CYP19A1 in BT-474 cells could significantly affect the activity of breast cancer cells (Figure 1D). Overexpression of CYP19A1 reduced the IC50 value of BT-474 pairs of Letrozole from 16923±45.33 nM to 7429±52.00 nM, while interference of CYP19A1 increased the IC50 value of BT-474 pairs of Letrozole from 867.6±64.07 nM to 1900±54.75 nM (Figure 1E). These results suggest that low expressed CYP19A1 was associated with letrozole resistance in ER-positive breast cancer cells.

### MiR-19a-3p is upregulated in ER positive breast cancer

**Figure 2:**
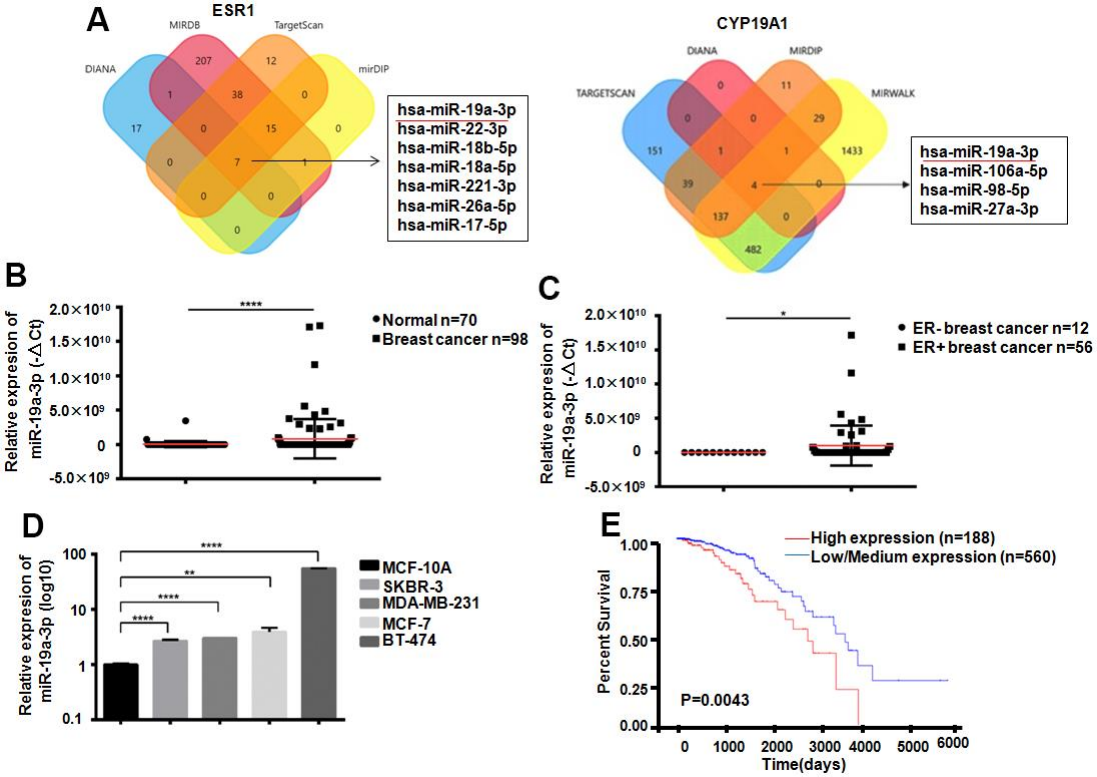
**Expression of miR-19a-3p in breast cancer.** (**A**) Bioinformatics analysis showed that miR-19a-3p could target ESR1 and CYP19A1. (**B**) The expression levels of miR-19a-3p in plasma of 70 normal women and 98 breast cancer patients were detected by RT-QPCR, using U6 as internal reference and analyzed by Mann Whitney test.****P<0.0001. (**C**) The expression levels of miR-19a-3p in plasma of 12 patients with ER negative breast cancer and 56 patients with ER positive breast cancer were detected by RT-QPCR, using U6 as internal reference and analyzed by Mann Whitney test.*P<0.05. (**D**) RT-QPCR was used to detect the expression levels of miR-19a-3p in normal breast epithelial cells and 4 breast cancer cell lines. With U6 as internal reference, 3 replicates were performed for 3 times each time. **P<0.01, ****P<0.0001. (**E**) Kaplan-Meier curve of overall survival of breast cancer patients with low expression (n = 560) and high expression (n = 188) of miR-19a-3p.

ESR1as a gene which could encoding ER. Its expression was closely related to Letrozole resistance in breast cancer therapy Through analysis of 4 databases, 7 miRNAs that can target ESR1 were screened out, and 4 miRNAs that can target CYP19A1 were screened out through bioinformatics analysis. Venn diagram showed that miR-19a-3p could bind both ESR1 and CYP19A1 targetly (Figure 2A). Compared with 70 normal women, 98 breast cancer patients exist miR-19a-3p overexpressed in plasma, ***P<0.001 (Figure 2B). In addition, we also found that the expression of miR-19a-3p was elevated both in the plasma of ER-positive breast cancer patients and in ER-positive breast cancer cells (Figure 2C, 2D). According to TCGA database analysis, the higher expression level of miR-19a-3p in breast cancer with the lower the survival rate (*P*=0.0043).

### miR-19a-3p inhibits the sensitivity of ER positive breast cancer to Letrozole

**Figure 3:**
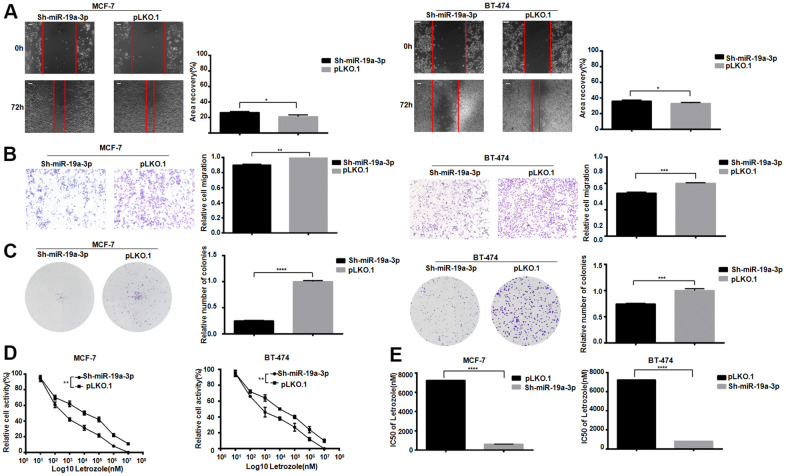
**Knockdown of miR-19a-3p decreases the IC50 of Letrozole in breast cancer cells.** (**A**) Cell healing rate at 0 hours and 72 hours was measured by scratch assay with the scale of 100 μm. The experiment was repeated 3 times.*P<0.05. (**B**) Transwell experiment was performed to detect the effect of knockdown miR-19a-3p on the mobility of breast cancer cells. The experiment was repeated for 3 times with 20× microscope field.**P<0.01 and ***P<0.001. (**C**) Cell clonal formation assay was performed to detect the effect of knockdown miR-19a-3p on breast cancer cell colony formation. The experiment was repeated for 3 times.***P<0.001, ****P<0.0001. (**D**) CCK-8 was used to detect cell activity, and each group had 3 repeated wells.**P<0.01. (**E**) Graphpad Prism calculated IC50 of MCF-7 and BT-474 cells against Letrozole. ****P<0.0001.

After knockdown the miR-19a-3p in MCF-7 and BT-474, scratch test results showed that cell healing rate was inhibited in the experimental group (Figure 3A). The experimental group showed the migration was suppressed in MCF-7 and BT-474 (Figure 3B). Lower miR-19a-3p expression reduced colony formation in MCF-7 and BT-474 cell (Figure 3C). After treated by 8 concentrations Letrozole for 48 hours drug incubation, the CCK-8 activity was used to detect the miR-19a-3p affected in MCF-7 and BT-474 (Figure 3D). MiR-19a-3p knocked down reduced the IC50 value of MCF-7 pair of Letrozole from 7231±51.50 nM to 621.1±64.73 nM, and knocking miR-19a-3p reduced the IC50 value of BT-474 pair of Letrozole from 7231±51.50 nM to 810.5±62.07 nM (Figure 3E). Indicated that the high expression of miR-19a-3p promoted the letrozole resistance in ER-positive breast cancer.

### LINC00094 targets miR-19a-3p in breast cancer cells

**Figure 4:**
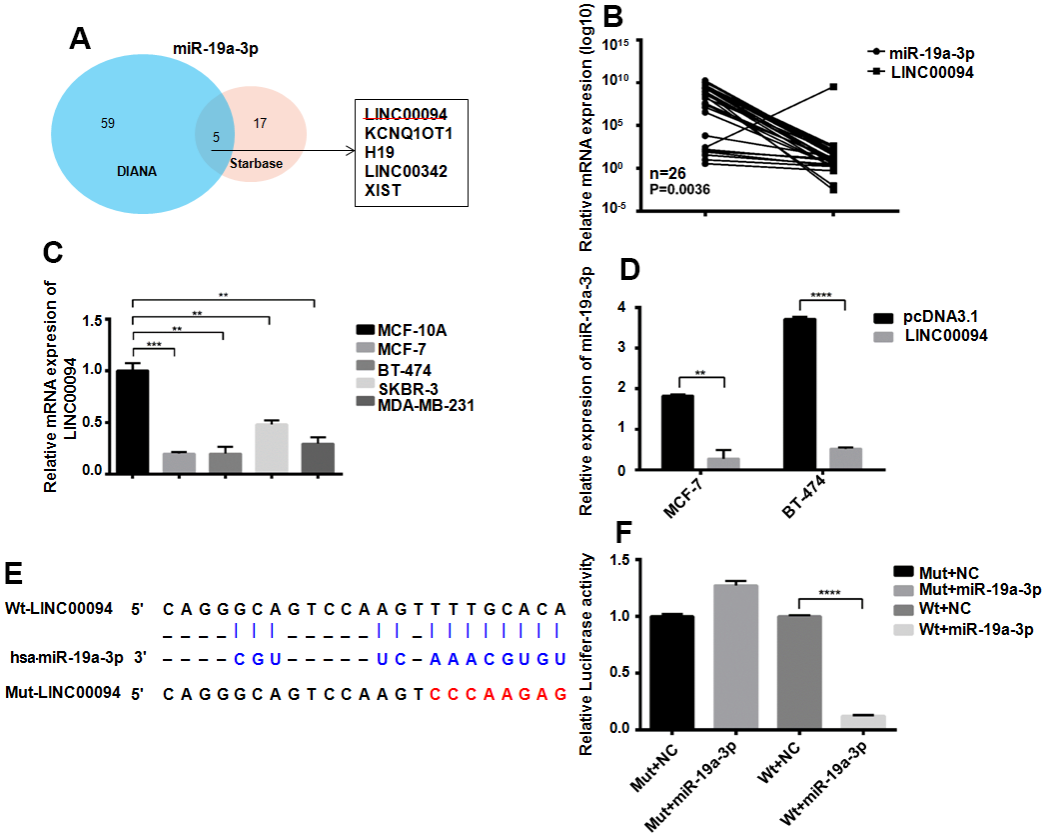
**Adsorption of miR-19a-3p by LINC00094 sponge in breast cancer.** (**A**) Bioinformatics analysis of LINCRNA binding to miR-19a-3p. (**B**) RT-QPCR was used to detect the expression levels of miR-19a-3p (U6 as internal reference) and LINC00094 (β-actin as internal reference) in plasma of breast cancer patients. (**C**) Endogenous expression of LINC00094 in breast cancer cells was detected by RT-QPCR. *P<0.05, **P<0.01, ***P<0.001. (**D**) miRNA specific PCR detection of miR-19a-3p expression level (U6 as internal reference). **P<0.01, ****P<0.0001. (**E**) Binding sequence of miR-19a-3p and LINC00094, and mutation site of mutant LINC00094. (**F**) Luciferase activity test of HEK-293T cells in each transfection group, ****P<0.0001.

The targeted lncRNAs of miR-19a-3p were detected by two bioinformatics analysis websites. 5 targeted lncRNAs to miR-19a-3p were screened out by Venn diagram (Figure 4A). RT-QPCR showed the downregulated of LINC00094 in 26 breast cancer patients plasma, while miR-19a-3p was overexpressed, P=0.0036 (Figure 4B). Compared with normal breast epithelial cells, LINC00094 was lower expressed in the 4 types of breast cancer cells (Figure 4C) However, high expression of miR-19a-3p in those cells. Overexpression of LINC00094 in MCF-7 and BT-474 cells significantly reduced the miR-19a-3p level (Figure 4D). MiR-19a-3p binds to the cDNA sequence of LINC00094, and luciferase activity test results show that, after co-transfection of wild-type plasmids pcDNA3.1-LINC00094 and overexpressed plasmids pLKO.1-miR-19a-3p. The luciferase activity of pLKO.1-miR-19a-3p-HEK-293T cells was significantly reduced (Figure 4E, 4F).

### LINC00094 promotes sensitivity of ER positive breast cancer to Letrozole

**Figure 5:**
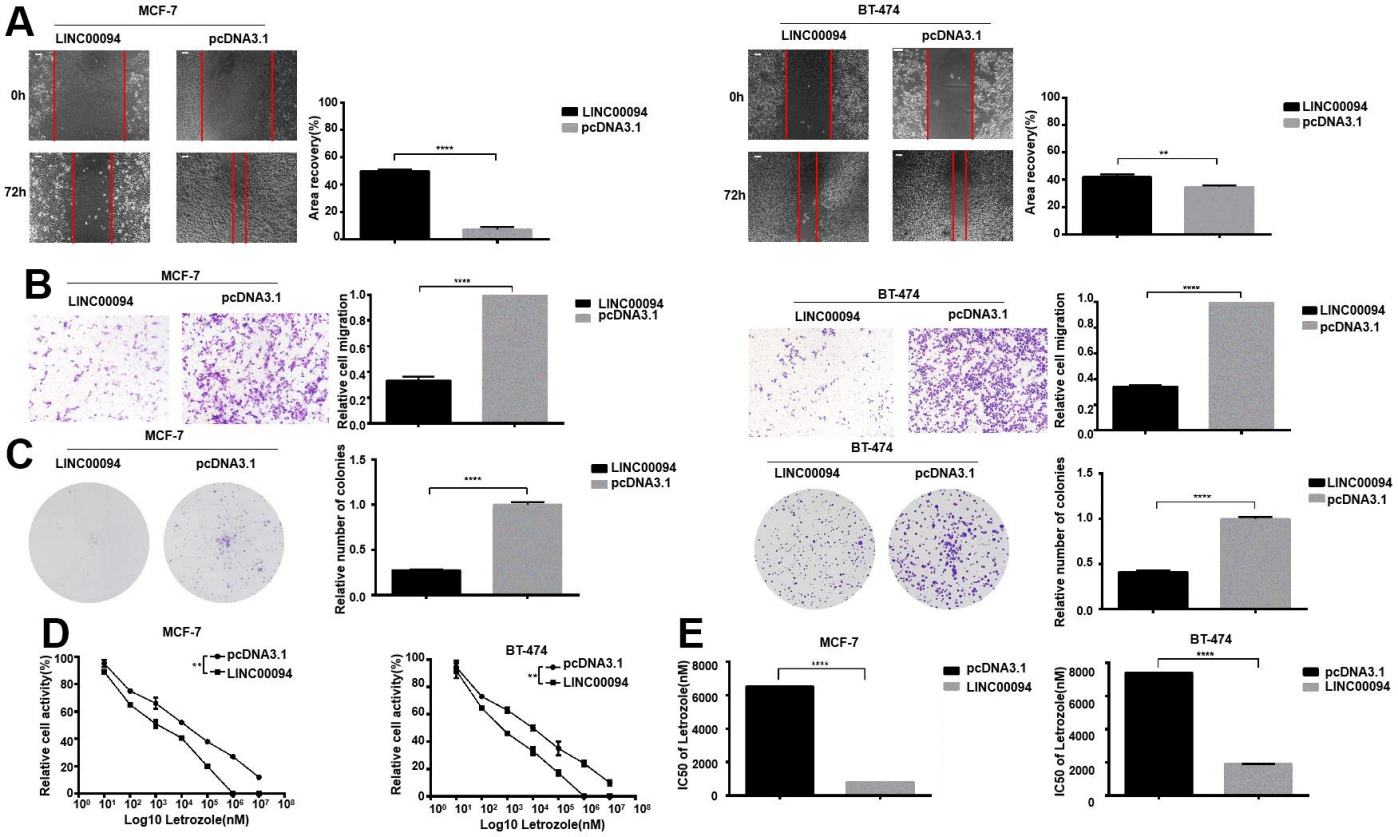
**Overexpression of LINC00094 affects IC50 of breast cancer cells to Letrozole.** (**A**) The cell healing rate at 0 hours and 72 hours was measured by scratch assay with the scale of 100 μm. The experiment was repeated 3 times. **P<0.01, ****P<0.0001. (**B**) Transwell assay was performed to detect the effect of LINC00094 overexpression on the mobility of breast cancer cells, 20× microscope field, and the experiment was repeated 3 times. ****P<0.0001. (**C**) Cell clonal formation assay was performed to detect the effect of overexpression of LINC00094 on the colony formation of breast cancer cells. The experiment was repeated for 3 times.****P<0.0001. (**D**) CCK-8 was used to detect cell activity, and each group had 3 repeated wells.**P<0.01. (**E**) Graphpad Prism calculated IC50 of MCF-7 and BT-474 cells against Letrozole. ****P<0.0001.

LINC00094 was overexpressed in MCF-7 and BT-474, and the scratch assay showed that the experimental group cells had lower migration ability compared with control group (Figure 5A, 5B). Overexpression of LINC00094 reduced the formation of BT-474 and MCF-7 cell colonies (Figure 5C). In BT-474 and MCF-7, after treated by 8 concentrations Letrozole for 48 hours in all group, the CCK-8 activity test showed that the LINC00094 overexpression reduced the cell activity of MCF-7 and BT-474 (Figure 5D). Interestingly, overexpression of LINC00094 reduced the IC50 value of MCF-7 versus Letrozole from 7429±51.75 nM to 1871±61.08 nM, and the IC50 value of BT-474 versus Letrozole from 6538±52.92 nM to 807.1±66.34 nM (Figure 5E). Our results suggest that the overexpressed of LINC00094 can inhibit the letrozole resistance in ER-positive breast cancer.

### The adsorption of miR-19a-3p by LINC00094 sponge affects the expression of CYP19A1

**Figure 6:**
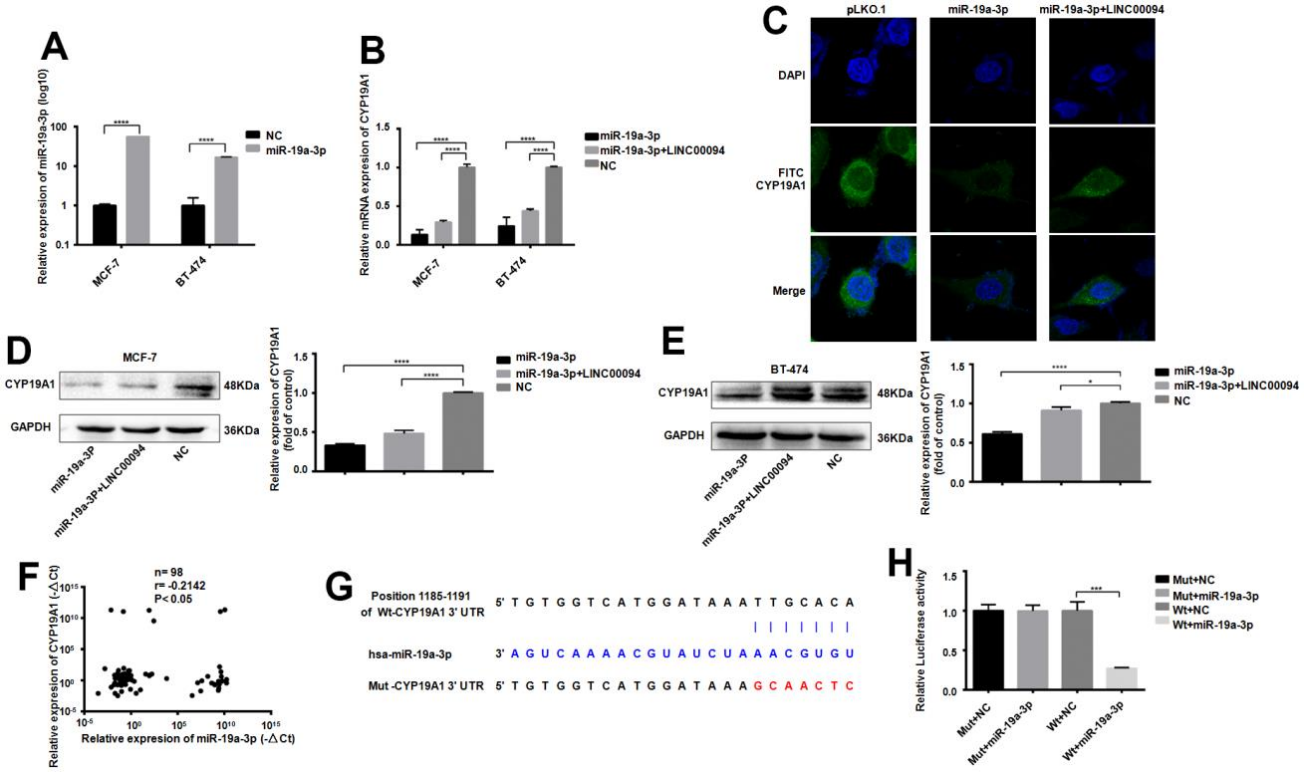
**Adsorption of miR-19a-3p by LINC00094 sponge affects the expression of CYP19A1.** (**A**) miRNA-specific PCR was used to detect the expression level of miR-19a-3p (U6 was an internal reference). ****P<0.0001. (**B**) mRNA expression of CYP19A1 in each transfection group of breast cancer cells was detected by RT-QPCR (β-actin as internal reference). ****P<0.0001. (**C**) Immunofluorescence analysis of DAPI and FITC-CYP19A1 expression in each transfection group of BT-474 cells, with the scale of 10 μm. (**D**) Western Blot was used to detect the protein expression level of CYP19A1 in each transfection group of MCF-7, with GAPDH as internal reference. The experiment was repeated for 3 times. ****P<0.0001. (**E**) Western Blot was used to detect the protein expression level of CYP19A1 in BT-474 transfected cells, with GAPDH as internal reference, and the experiment was repeated for 3 times. *P<0.05, ****P<0.0001. (**F**) The expression levels of CYP19A1 (β-actin as internal reference) and miR-19a-3p (U6 as internal reference) in plasma of breast cancer patients were detected by RT-QPCR. *P<0.05. (**G**) Binding sequence of miR-19a-3p to CYP19A1 3’UTR and mutation site of mutant CYP19A1 3’UTR plasmid. (**H**) Luciferase activity assay of HEK-293T cells in each transfection group. ***P<0.001.

The successful transfection of miR-19a-3p in MCF-7 and BT-474 cells significantly reducedCYP19A1 mRNA expression, while overexpressed of LINC00094 and miR-19a-3p promote the effect of CYP19A1 (Figure 6A, 6B). Intracellular CYP19A1 expression was detected by immunofluorescence, showing that the fluorescence expression level of FITC of CYP19A1 was reduced after overexpressed miR-19a-3p in BT-474. Moreover, overexpressed LINC00094 partially increased the fluorescence expression level of FITC of CYP19A1 compared with experimental group (Figure 6C). When miR-19a-3p was transfected into MCF-7 and BT-474 cells, the protein CYP19A1 expression was downregulated in overexpressed miR-19a-3p group significantly. Co-transfected with miR-19a-3p and LINC00094, CYP19A1 protein level was partially upregulated compared with miR-19a-3p alone (Figure 6D, 6E). RNA-seq of 98 breast cancer patients showed that the high expression of CYP19A1 was correlated with the low expression of miR-19a-3p (Figure 6F). miR-19a-3p target the 3’UTR sequence to CYP19A1; Results of luciferase activity experiment was showed by the luciferase activity of HEK-293T cells. These results showed that luciferase activity was significantly declined after co-transfection of pmirGLO-CYP19A1 3’UTR wild-type plasmid and pLKO. 1- miR-19a-3p overexpressed plasmid (Figure 6G, 6H).

### The LINC00094/ miR-19a-3p /CYP19A1 axis affects EMT progression in breast cancer

**Figure 7:**
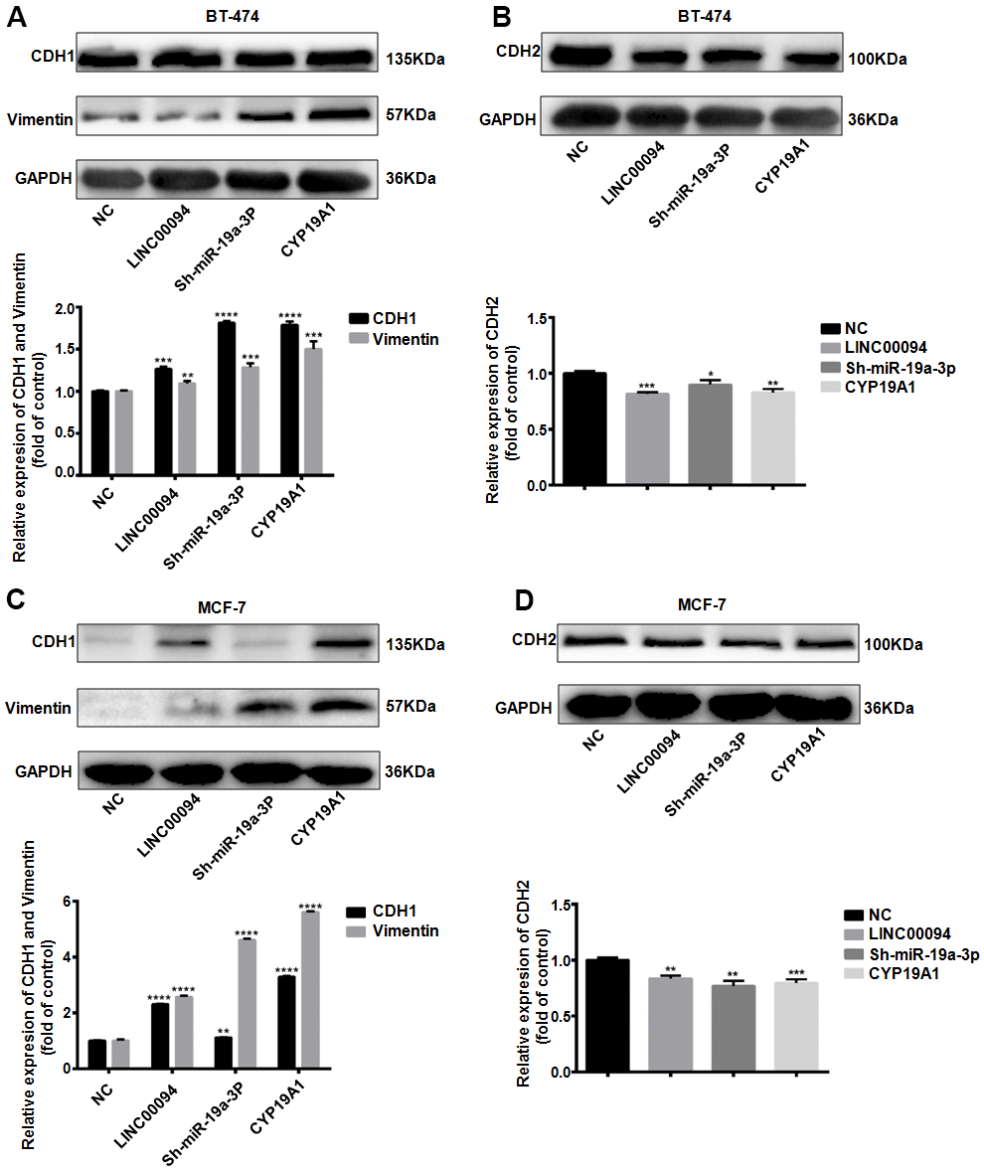
**The LINC00094/ miR-19a-3p /CYP19A1 axis affects EMT progression in breast cancer.** (**A**) Western Blot was used to detect the protein expression levels of CDH1 and Vimentin in BT-474 transfected cells, with GAPDH as internal reference. The experiment was repeated for 3 times. **P<0.01, ***P<0.001, ****P<0.0001. (**B**) Western Blot was used to detect the protein expression level of CDH2 in BT-474 transfected cells, with GAPDH as internal reference. The experiment was repeated for 3 times. * *P* <0.05, **P<0.01, ***P<0.001, ****P<0.0001. (**C**) Western Blot was used to detect the protein expression levels of CDH1 and Vimentin in MCF-7 transfected cells, with GAPDH as internal reference. The experiment was repeated for 3 times. **P<0.01, ****P<0.0001. (**D**) Western Blot was used to detect the protein expression level of CDH2 in MCF-7 transfected cells, with GAPDH as internal reference. The experiment was repeated for 3 times. **P<0.01, ***P<0.001.

When tumors promote cancer cell invasion and metastasis through the EMT pathway, CDH1 and Vimentin are often downregulated, while CDH2 is overexpressed. In BT-474, Immunoblotting indicated that the CDH1 and Vimentin were overexpressed in the three transfection groups, while the expression levels of CDH2 decreased (Figure 7A, 7B). Further immunoblotting in MCF-7 showed the expression levels of CDH1 and Vimentin in the three transfection groups were increased, while the expression levels of CDH2 decreased (Figure 7C, 7D).

### *In vivo* studies demonstrated the role of LINC00094/ miR-19a-3p in breast cancer

**Figure 8:**
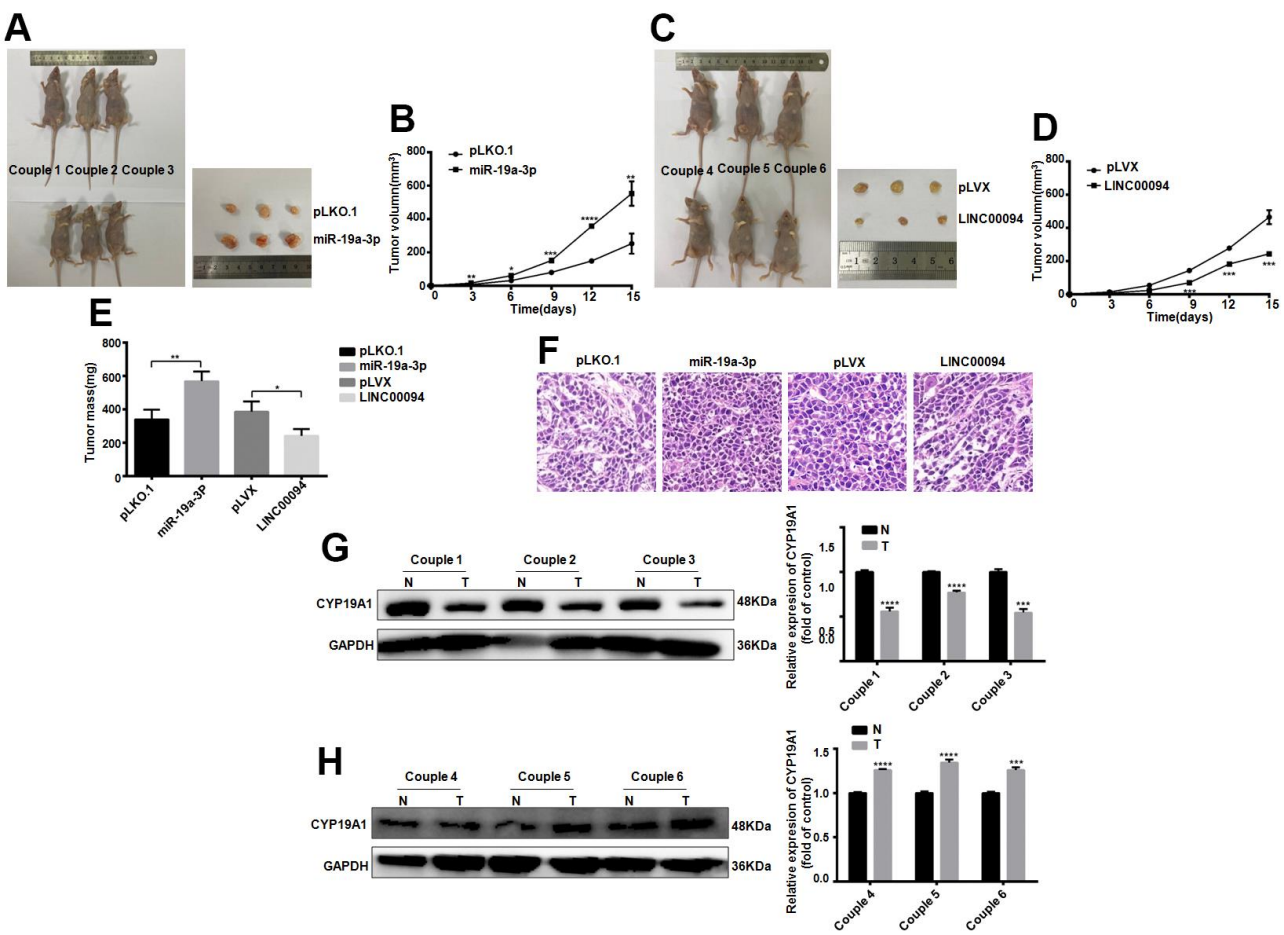
**Tumorigenesis experiments in nude mice *in vivo* proved the effects of LINC00094 and miR-19a-3p on breast cancer.** (**A**) 5×10^6^ BT-474 cells with stable expression of miR-19a-3p were injected into the breast fat pad of 4-week-old female BALB/c nude mice, and tumors were collected on the 15th day of tumor formation. (**B**) The tumor size of nude mice was measured every 3 days. (**C**) 5×10^6^ BT-474 cells statically expressed by LINC00094 were injected into the breast fat pad of 4-week-old female BALB/c nude mice, and tumors were collected on the 15th day of tumor formation. (**D**) Tumor size of nude mice was measured every 3 days. 3-5 nude mice of LINC00094 stable expression group and control group were respectively. There was statistical difference between experimental group and control group. ***P<0.001. (**E**) On the 15th day, the tumor of nude mice in each group was taken and weighed. There were statistical differences between the experimental group and the control group. *P<0.05, **P<0.01. (**F**) Tumor tissues of nude mice were stained with HE. (**G**) Western Blot was used to detect CYP19A1 protein expression level of the first pair to the third pair, with GAPDH as internal reference, and the experiment was repeated for 3 times. N is the control group. T is the experimental group. ***P<0.001, ****P<0.0001. (**H**) Western Blot was used to detect CYP19A1 protein expression level of the 4th to 6th pairs, with GAPDH as internal reference, and the experiment was repeated for 3 times. N is the control group. T is the experimental group. ***P<0.001, ****P<0.0001.

Next, the effect of LINC00094 and miR-19a-3p on oncogenesis in breast cancer was detected *in vivo*. Cell lines with stable and high expression of LINC00094 and miR-19a-3p and their control group were injected into the fat pad of the fourth pair of left breasts of 28 days female BALB/c nude mice. The tumor growth rate and volume were increased significantly in miR-19a-3p stable overexpression group (Figure 8A, 8B). On the contrary, the tumor growth rate and tumor volume in the stable LINC00094 overexpression group of were reduced (Figure 8C, 8D). As well as the tumor body weight increased in miR-19a-3p overexpression group (Figure 8E). HE staining analysis of tumor tissues in nude mice showed more mitotic cells in the stable miR-19a-3p overexpression group, while the mitotic count decreased in the LINC00094 stable overexpression group (Figure 8F). In the miR-19a-3p stable overexpression group, the expression level of CYP19A1 was significantly decreased. IHC staining of CYP19A1 was increased (Figure 8G, 8H). Overall, these *in vivo* tumorigenic suggest that miR-19a-3p expression promotes breast tumor growth, while LINC00094 expression inhibits breast cancer growth.

## DISCUSSION

Studies have found that in addition to ESR1 and CYP19A1 gene mutations, 21.5% of AIs drug-resistant breast cancer patients have abnormal amplification of CYP19A1 gene [31]. In our study, we first determined that the expression of CYP19A1 in the plasma of drug-resistant breast cancer patients was significantly upregulated, indicated that the effect of CYP19A1 on drug sensitivity in breast cancer may be bidirectional.

MiR-19a-3p as one of miR-17-92 family, four highly conserved seed regions exist in other members (miR-17, miR-18a, miR-20a and miR-92a) [32]. MiR-17-92 family, as a multifunctional oncogenic miRNAs, had closely relationship with tumor angiogenesis and development [33, 34]. Our previous studies had detected the miR-93-5p could inhibit breast cancer by regulating STAT3 signaling pathway [11, 12]. Another research indicated miR-19a-3p could promote the osteosarcoma and lung cancer cells malignancy through the EMT pathway [35–37]. RNA-seq of clinical tumor samples show that miR-19a-3p was downregulated in ER-positive breast cancer patients plasma, with lower miR-19a-3p expression, the highly proliferation and migration by and EMT pathway can be detected in MCF-7 and BT-474cells. These researches suggested miR-19a-3p expression level may become a prognostic marker for RE-positive breast cancer patients. However, the mechanism to downregulate the miR-19a-3p in ER negative breast cancer patients is unclear. Previous studies had shown that lncRNAs can sponge the downstream miRNAs then exert their function. LINC00094 is a newly discovered LncRNA. In Alzheimer’s disease, combined with MEM, silencing LINC00094 may provide a new therapy target [30]. But targeting to LINC00094 to improve the Letrozole therapy sensitivity in ER positive breast cancer is unclear. Overall, we found that the expression level of LINC00094 was closely associated with ER-positive breast cancer with highly expression of miR-19a-3p in plasma was closely related to the overall survival rate in breast cancer patients. Targeting LINC00094 can inhibited the migration and EMT process of MCF-7 and BT-474 cells. Mechanistically, LINC00094 could sponge adsorbed miR-19a-3p. LINC00094 may be a new strategy for breast cancer which resistance the AIs. These findings reveal an important regulatory mechanism of letrozole resistance in breast cancer cells.

## MATERIALS AND METHODS

### Plasmids

The coding region sequences of CYP19A1 genes were amplified by PCR using the cDNA of BT-474 as the template (CYP19A1: forward: 5’-TGCTGGATATCTGCAGAATTCATGGTTTTGGAAATGCTGAACC-3’, reverse: 5’-TAGTCCAGTGTGGTGGAATTCCTAGTGTTCCAGACACCTGTCTGAGT-3’) and inserted into pcDNA3.1 (Invitrogen, USA). The sequences of LINC00094 genes were amplified by PCR using the cDNA of BT-474 as the template (LINC00094 forward: 5’-TGCTGGATATCTGCAGAATTCGCAGTGCGTCGCGCC-3’, reverse: 5’-TAGTCCAGTGTGGTGGAATTCCCGCCTTGCTGTGGGAGT-3’) and inserted into pcDNA3.1 (Invitrogen, USA). The CYP19A1 3’-UTR sequence was amplified by PCR (Wt-CYP19A1 forward: 5’-TGTTTAAACGAGCTCGCTAGCAGAAGGCTGGTCAGTACCCA-3’, CYP19A1 reverse: 5’-GACTCTAGACTCGAGGCTAGC TACTTTGACAAGGTTTAATTAGTATGTC-3’) and Mut-CYP19A1 forward 1: 5’-TTTAAACGAGCTCGCTAGCCAGAAGGCTGGTCAGTACCCA-3’, Mut-CYP19A1 reverse 1: 5’-TTAGAAAGCGAATTCCAAGGACACGTTTTTATCCATGACCACATACA-3’; Mut-CYP19A1 forward 2: 5’-TGTATGTGGTCATGGATAAAAACGTGTCCTTGGAATTCGCTTTCTAA-3’, Mut-CYP19A1 reverse 2: 5’-CAGGTCGACTCTAGACTCGATACTTTGACAAGGTTTAATT-3’), which inserted into pmirGLO (Invitrogen, USA). The LINC00094 sequence was amplified by PCR (Wt-LINC00094 forward: 5’-TGTTTAAACGAGCTCGCTAGCGCAGTGCGTCGCGCC-3’, Wt-LINC00094 reverse: 5’-GACTCTAGACTCGAGGCTAGCCCGCCTTGCTGTGGGAGT-3’) and (Mut-LINC00094 forward 1: 5’-TGTTTAAACGAGCTCGCTAGCGCAGTGCGTCGCGCCGCT-3’, Mut-LINC00094 reverse 1: 5’-TCACATCGGAATTACATAATCTCTTGGGACTTGGACTGCCCTGTGTCC-3’; Mut-LINC00094 forward 2: 5’-GGACACAGGGCAGTCCAAGTCCCAAGAGATTATGTAATTCCGATGTGA-3’, Mut-LINC00094 reverse 2: 5’-GACTCTAGACTCGAGGCTAGCCCGCCTTGCTGTGGGAGT-3’), which inserted into pmirGLO (Invitrogen, USA). The stable knockdown miR-19a-3p sequence was amplified by PCR (miR-19a-3p–shRNA: forward: 5’-CACCGGTAATGGGTCAACTGAAACACGAATGTTTCAGTTGACCCATTACC-3’, miR-19a-3p–shRNA: reverse: 5’-AAAAGGTAATGGGTCAACTGAAACATTCGTGTTTCAGTTGACCCATTACC-3’) and inserted into pLKO.1 (Invitrogen, USA). The stable overexpression miR-19a-3p sequence was amplified by PCR (miR-19a-3p forward: 5’-CCGGGCAGTCCTCTGTTAGTTTTGCATAGTTGCACTACAAGAAGAATGTAGTTGTGCAAATCTATGCAAAACTGATGGTGGCCTGC-3’, miR-19a-3p reverse: 5’-AATTGCAGGCCACCATCAGTTTTGCATAGATTTGCACAACTACATTCTTCTTGTAGTGCAACTATGCAAAACTAACAGAGGACTGC-3’) and inserted into pLKO.1 (Invitrogen, USA). The stable overexpression LINC00094 sequence was amplified by PCR (LINC00094 forward: 5’-GAGGATCTATTTCCGGTGAAGCAGTGCGTCGCGCC-3’, LINC00094 reverse: 5’-CTAGAACTAGTCTCGAGGAACCGCCTTGCTGTGGGAGT-3’) and inserted into pLVX (Invitrogen, USA). The PCR fragment was connected with the vector into plasmid by homologous recombination kit (Vazyme, China).

### RT-QPCR

Total RNA was extracted using TRIzol (Vazyme, China). 1ugRNA was reverse transcribed into cDNA(Vazyme, China), and SYBR-green (Vazyme, China) reaction system was used for qRT-PCR. The following primers were used:

β-actin: forward: 5′-CCTTCCTGGGCATGGAGTC-3′, reverse: 5′- TGATCTTCATTGTGCTGGGTG-3′.

CYP19A1: forward: 5′-CTGCCGAATCGAGAGAGCTGTA-3′, reverse: 5′-ACGGCAGATTCCTGTGGATG-3′.

miR-19a-3p-RT:5′-GTCGTATCGACTGCAGGGTCCGAGGTATTCGCAGTCGATACGACTCAGTT-3′.

miR-19a-3p: forward: 5′-CGGCTGTGCAAATCTATGCAA-3′

common: reverse: 5′-ACTGCAGGGTCCGAGGTATT-3′

LINC00094: forward: 5′-ACTGAAAAAGCTGGCACTGGG-3′, reverse: 5′-GCGTTTGTTCAGGTCTCGTCT-3′.

### Cell culture

Human normal mammary epithelial cells MCF-10A, human breast cancer cells MDA-MB-231, SKBR-3, BT-474 and MCF-7 were purchased from the cell bank of typical culture preservation Committee of Chinese Academy of Sciences. BT-474 cells were cultured in RPMI1640 (GIBCO, USA) medium, MDA-MB-231, SKBR-3 and MCF-7 cells were cultured in DMEM medium. MCF-10A was cultured in special medium (Servicebio, China). All cells were cultured in the medium supplemented with 10% fetal bovine serum (GIBCO, USA), at 37° C and 5% CO_2_.

### Cell transfection and lentiviral transfection

Lipofectamine 2000 was used for transfection of all cells (Vazyme, China). The HEK-293T cells were co-transfected null vector pLKO.1 or pLVX or miR-19a-3P-pLKO.1 or LINC00094-pLVX with VSVG and GAG-POL for virus packaging. The virus was harvested at 24-48 hours after transfection. The concentrated virus was dropped into the cell line, and puromycin (Sigma, USA) was used to select the successfully infected cell line.

### Western blot

Western blot analysis was performed as described previously [11]. The antibodies used are as follows: GAPDH (1:5000, Proteintech Group, USA), CYP19A1 (1:1000, ABclonal, China), CDH1 (1:2000, CST, USA), CDH2 (1:2000, CST, USA), Vimentin (1: 2000, CST, USA).

### Wound healing assay

Briefly,1×10^5^ cells were inoculated in 6-well plates. After 24 hours, a 200 μL pipette was gently scratched on the surface of the cells. Pictures were taken under an inverted microscope (Olympus, Japan) at 0 and 72 hours.

### Migration and invasion analysis

Corning’s Transwell Chamber (Corning, USA) was used to analysis the migration and invasion. Serum-free medium containing 5×10^5^ cells was added to the upper chamber, and after 36 hours of incubation, and after 36 hours of culture, cells were stained and photographed and analyzed with a microscope.

### Analysis of cell colony formation

1×10^3^ cells were seeded in 6-well plates, the medium was changed every 3 days, fixed with paraformaldehyde one week later, and stained with crystal violet. Microscopic observation and counting of cell colonies (Olympus, Japan).

### Cell viability analysis

Inoculated into the plate medium in 96-well plate, 1×10^4^ cells/100 μL per well. After the cells were adhere to the well, different concentrations of Letrozole (Meilunbio, China) were added to each group. After incubation with Letrozole for 48 hours, add 10 μL of CCK-8 solution (Beyotime, China). Cells were incubated in an incubator for 90 minutes and assayed at a wavelength of 490 nm (BioTek, USA).

### Luciferase analysis

HEK-293T cells were inoculated into 24-well plates, and then the wild-type or mutant recombinant plasmid and control plasmid were transfected into HEK-293T cells. There were 5 multiple wells in each group and 300 μL of medium was added in each well. At 37° C and 5% CO_2_ for 24 hours, the cells were lysed with 100 μL of lysate for 15 minutes (Meilunbio, China). After centrifugation, 10 μL supernatant was added to a 96-well plate containing 100 μL BCA protein quantification solution for protein quantification (Beyotime, China).

### Immunofluorescence and laser confocal imaging

The treated cells were sequentially fixed and permeabilized. Cells were incubated with CYP19A1 antibody (ABclonal, China) overnight at 4° C. FITC combined with anti-mouse IgG (Abcam) was used as secondary antibody. Laser confocal observation and photography (Olympus, Japan).

### Human tumor xenograft model

4-week-old female BALB/c nude mice were purchased from Beijing Huafukang Laboratory Animal Co., Ltd. 5 × 10^6^ cells were injected into the left fat pad of the fourth pair of breasts of BALB/c nude mice. Mice were sacrificed 15 days later for protein analysis and tumor histopathology.

### Collection and testing of plasma samples from patients

Plasma samples from all patients are retained specimens and no ethical approval is required. The expression levels of CYP19A1, miR-19a-3p and LINC00094 in plasma samples were detected by RT-QPCR. Among 98 patients with breast cancer, 26 were drug resistant and 72 were drug sensitive. There were 56 patients with ER positive breast cancer and 12 patients with ER negative breast cancer.

### Statistics

SPSS 17.0 and GraphPad Prism 7.0 were used for univariate and multivariate analysis of the experimental data. Statistical analysis was determined by two-tailed T-test or Mann Whitney test. The asterisk indicates statistical significance (*P<0.05; **P < 0.01; ***P<0.001, ****P<0.0001). Unless a mean ±SEM is specified in the legend, the data is reported as mean ±SD. Image J was used to make the quantitative graph of scratch experiment, Transwell experiment, clone formation experiment and Western blot experiment.

### Availability of supporting data

The data generated during this study are included in this article and its supplementary information files are available from the corresponding author on reasonable request.

### Ethics approval and consent to participate

All mouse experimental procedures and procedures were evaluated and authorized in strict accordance with the guiding principles of the Animal Protection and Use Committee of Wuhan University of Science and Technology and in accordance with the “Hubei Province Experimental Animal Management Regulations.”

### Consent for publication

All authors have read this manuscript and approved for submission.
